# Effect on Growth Performance and Nutritive Value of Cultivated *Azolla filiculoides* As An Alternative Feedstuff for Ruminant

**DOI:** 10.21315/tlsr2024.35.3.12

**Published:** 2024-10-07

**Authors:** Mohammad Fitri Rimi Hamidan, Mohd Noor Hisham Mohd Nadzir, Shamarina Shohaimi, Habsah Bidin, Noraini Samat

**Affiliations:** 1 Department of Biology, Faculty of Science, Universiti Putra Malaysia, 43400 UPM Serdang, Selangor, Malaysia; 2 Livestock Science Research Centre, MARDI Headquarters, 43400 Serdang, Selangor, Malaysia

**Keywords:** *Azolla filiculoides*, Growth Performance, Nutrient Composition, Digestibility, Ruminant, *Azolla filiculoides*, Prestasi Pertumbuhan, Komposisi Nutrien, Ketercernaan, Ruminan

## Abstract

*Azolla filiculoides* is a tiny, free-floating aquatic fern and has a potential alternative protein and fibre source for ruminants, was investigated for its cultivation optimisation and feedstuff suitability. Study 1 was conducted to investigate the influence of different fertiliser types (control, broiler manure, sheep manure, cow manure) and concentrations (0.25 g/L–1.25 g/L) on the growth performance (fresh weight, doubling time, relative growth rate) and nutrient composition (dry matter, ash, crude protein, crude fibre, crude fat) of *A. filiculoides*. The optimised type of fertiliser and concentration in Study 1 were further adopted in Study 2 to evaluate the effect of different fertiliser processing methods on the growth performance, nutritive value and *in vitro* rumen digestibility of *A. filiculoides* upon cultivation. The findings in Study 1 showed that cultivation of *A. filiculoides* using sheep manure at the concentration of 1.00 g/L is the best resulted in the shortest doubling time (3 to 5 days) and produced fresh weight (FW), relative growth rate (RGR), crude protein (CP) and crude fibre (CF) at 132.2 g/m^2^, 0.32 g/g/day, 21.2% DM^−1^ and 14.4% DM^−1^, respectively. Furthermore, unprocessed sheep manure (T3) exhibited superior (*p* < 0.05) fresh weight, relative growth rate, nutrient composition and fibre components compared to the burned manure treatment (T2). *In vitro* digestibility analysis discovered that T3 achieved a 24-hour accumulated gas production of 86.9 mL DM^−1^, with *in vitro* dry matter digestibility (IVDMD), *in vitro* organic matter digestibility (IVOMD) and metabolisable energy (ME) of 82.9%, 43.7% and 5.8 MJ/kg DM, respectively. These findings suggest that *Azolla filiculoides* cultivation can be economically optimised using 1.00 g/L unprocessed sheep manure (fresh manure), potentially serving as a self-produced, nutritious feedstuff for ruminants.

## Abstract (fr)

**Abstrak:**

*Azolla filiculoides* adalah paku pakis akuatik yang kecil dan terapung bebas dan mempunyai potensi sumber protein dan serat alternatif untuk ruminan, telah dikaji untuk penanaman optimum dan kesesuaian sebagai bahan makanan. Kajian 1 telah dijalankan untuk mengkaji pengaruh jenis baja yang berbeza (kawalan, tinja ayam daging, tinja biri-biri, tinja lembu) dan kepekatan (0.25 g/L–1.25 g/L) terhadap prestasi pertumbuhan (berat segar, masa penggandaan, kadar pertumbuhan relatif) dan komposisi nutrien (bahan kering, abu, protein kasar, serat kasar, lemak mentah) *A. filiculoides*. Jenis baja dan kepekatan yang dioptimumkan dalam Kajian 1 seterusnya diguna pakai dalam Kajian 2 untuk menilai kesan kaedah pemprosesan baja yang berbeza ke atas prestasi pertumbuhan, nilai pemakanan dan kebolehcernaan rumen *in vitro A. filiculoides* semasa penanaman. Dapatan dalam Kajian 1 menunjukkan bahawa penanaman *A. filiculoides* menggunakan tinja kambing biri-biri pada kepekatan 1.00 g/L adalah yang terbaik dalam menghasilkan masa penggandaan terpendek (3 hingga 5 hari) dan berat segar (FW), kadar pertumbuhan relatif (RGR), protein kasar (CP) dan gentian kasar (CF) masing-masing pada 132.2 g/m^2^, 0.32 g/g/hari, 21.2% DM^−1^ dan 14.4% DM^−1^. Selain itu, tinja kambing biri-biri yang tidak diproses (T3) menghasilkan berat segar yang terbaik (*p* < 0.05), kadar pertumbuhan relatif, komposisi nutrien dan komponen gentian berbanding rawatan tinja yang dibakar (T2). Analisis kebolehcernaan *in vitro* mendapati bahawa T3 mencapai pengeluaran gas terkumpul sebanyak 86.9 mL DM^−1^ dalam masa 24 jam, dengan kebolehcernaan bahan kering *in vitro* (IVDMD), kebolehcernaan bahan organik *in vitro* (IVOMD), dan tenaga boleh dimetabolismekan (ME) masing-masing sebanyak 82.9%, 43.7% dan 5.8 MJ/kg DM. Penemuan ini mencadangkan bahawa penanaman *A. filiculoides* boleh dioptimumkan dari segi ekonomi menggunakan 1.00 g/L tinja kambing biri-biri yang belum diproses (tinja segar), yang berpotensi berfungsi sebagai bahan makanan berkhasiat yang dihasilkan sendiri untuk ruminan.

**Highlights**
- *Azolla filiculoides* could be applied as an alternative source of fibre and protein for ruminant.
- This species was able to be self-cultivated by the farmers with minimum cost and practical.
- *Azolla filiculoides* was highly digested by the ruminants.

## INTRODUCTION

Ruminant livestock production faces a growing challenge in securing sustainable and cost-effective protein sources. *Azolla filiculoides*, a free-floating aquatic fern with nitrogen-fixing capabilities, has emerged as a promising alternative feedstuff due to its rapid growth and high protein content (Miranda *et al*. 2020; Bhujel & Rizal 2022). Miranda *et al*. (2020) in their study suggested that *A. filiculoides* could be one of the alternative bio-phytoremediation agents for minerals such as phosphorus and potentially used to treat different types of wastewaters. Besides, this species was able to improve the pH, turbidity, chemical oxygen demand (COD) and biological oxygen demand (BOD) of dairy farm wastewater by decreasing nitrogen (N) and phosphorous (P) at 30.8% and 7.4%, respectively (Krishna *et al*. 2022). *Azolla filiculoides* shows potential as an eco-friendly feed option for raising ruminant livestock. Sustainability includes economic sustainability (Kollah *et al*. 2016), decreased competition with human food sources (Schader *et al*. 2015), and minimal land-use needs (Tallentire *et al*. 2018). Prior studies have shown that Azolla can be effectively used as a substitute protein source in poultry (Acharya *et al*. 2015), aquaculture (Shukla *et al*. 2018; Samad *et al*. 2020), and even monogastric herbivores like horses (Elghandour *et al*. 2018) and rabbits (Sireesha *et al*. 2017) as this species has also been reported as a high-protein plant and potentially used as an alternative feedstuff for animals (Mézes 2018; Ansal 2020; Bhatt *et al*. 2020; Nasir *et al*. 2022).

However, there is a report that mentions some of the negative implications of utilising the Azolla plant from wild sources. Wild Azolla can be contaminated with foreign materials and lack control over its nutritional profile (De Wet *et al*. 1990). Freshwater shrimp appeared to thrive or reside within the root system of the plant and therefore had become part of the harvested Azolla itself. Consequently, major implications such as decomposing shrimp will increase plant decomposition, reduce palatability to stock and provide media for mycotoxin growth which is toxic chemicals produced by various fungi such as *Aspergillus flavus, Aspergillus parasiticus, Penicillium expansum, Penicillium marneffei, Claviceps purpurea* and many more that will affect the marketability of this feedstuff. Restriction of Animal Health Australia outlines Restricted Animal Material (RAM) and the current Australian Ruminant Feed Ban which is an “inclusive ban on the feeding to all ruminants of all meals, including meat and bone meal derived from all vertebrates, including fish and birds”. Therefore, in this case, due to the presence of freshwater shrimp during the harvesting process, certain countries such as Australia did not allow Azolla feed to be fed to ruminant stock. This legislation extends to ruminants of all classes (Huggins 2007). Cultivation allows for optimised growth conditions and ensures consistent nutrient composition. Cultivating Azolla offers several advantages over utilising wild sources. Previous studies have demonstrated the potential of Azolla as a feed supplement for various livestock species (Acharya *et al*. 2015; Sireesha *et al*. 2017; Shukla *et al*. 2018; Mézes 2018; Ansal 2020; Bhatt *et al*. 2020; Samad *et al*. 2020; Nasir *et al*. 2022). Therefore, cultivating Azolla on the farm is a good practice to produce our source of protein and fibre for livestock. While phosphorus is crucial for optimal growth and Azolla production (Arora & Saxena 2005), simply using high concentrations of phosphorus would not necessarily result in the maximum nutrient content within Azolla itself (Handajani 2011). Furthermore, the unstable chemical input prices in the market will increase the production costs of Azolla and feed. Therefore, the use of livestock manures entirely as a source of nutrients in supporting the growth of Azolla is an effective alternative to complement an economical and practical ruminant feed formulation. As reported by Alhrout *et al*. (2018) which has proved the use of farm manure sewage was able to fulfil the nutrient requirements for plant growth. Studies have shown that *Azolla filiculoides* can exhibit rapid growth rates under optimal conditions. However, the specific yield can vary depending on factors like nutrient availability, light intensity, and water temperature (Golzary *et al*. 2021).

In the context of the common practice for farmers or farm workers, two aspects had to be emphasised which are practicality and applicability. Mostly farmers or livestock entrepreneurs will look at the cost and the efficiency of the technology and balance it with the return that they could get from the application in their farm. While raw manure can be used as a fertiliser for Azolla cultivation, it may require processing to improve its suitability. Drying manure can be expensive for small-scale farmers (Pandiyan *et al*. 2022). Burning manure, a traditional practice in some regions has been proposed as an alternative drying method. The burning process was an alternative procedure to remove excess moisture from the manure and increase the phosphorus content which improves the fertility level of the media and in turn, increases the growth rate and yield of crops (El Sobky 2017). However, burning manure can lead to significant losses of volatile nutrients, particularly nitrogen (Xie *et al*. 2015). Additionally, the high temperatures achieved during burning can convert some phosphorus into unavailable forms for plant uptake (Dail *et al*. 2007). In addition to achieving maximum production, the phosphorus (P) requirements for Azolla species such as *A. microphylla*, *A. pinnata* and *A. filioculoides* have been reported to be different, i.e., 2.0 ppm, 5.0 ppm and 20 ppm, respectively (Cary & Weerts 1992). This was in line with the study of Majumdar *et al*. (1993) suggested that phosphorus is important for the optimum growth of Azolla. However, the highest concentration does not result in the highest nutrient content such as protein (Handajani 2011). However, optimising Azolla cultivation for ruminant feed requires careful consideration of nutrient requirements and cost-effectiveness. This study investigates the influence of different manure-based fertilisers and their concentrations on the growth performance (fresh weight, doubling time, relative growth rate) and nutrient composition (dry matter, ash, crude protein, crude fibre, crude fat) of cultivated *A. filiculoides*.

## MATERIALS AND METHODS

### Experimental Site

The cultivation of *A. filiculoides* for Study 1 was conducted in controlled tanks at the Malaysia Agriculture Research and Development Institute (MARDI) Headquarters in Serdang, Selangor. Each tank was filled with clean water until it reached the overflow outlet before the cultivation process. Dichlorination was done by exposing all tanks to sunlight overnight to evaporate the existing chlorine into gases as described by Robidoux *et al*. (2018). This approach ensured precise monitoring of environmental factors such as water quality, nutrient availability, pH and temperature, which are crucial for optimising Azolla growth performance and nutrient composition. The cultivation period lasted for two weeks (14 days). This timeframe aligns with previous research indicating that *A. filiculoides* can achieve significant biomass under optimal conditions within two weeks (Stewart & Boyd 1999; Astuti & Indriatmoko 2018).

### Rumen *in vitro* Digestibility Study

Next, a digestibility trial for Study 2 was done at MARDI Kluang research station, Johor. Preparation of the digestibility trial assay was done at the Digestibility Laboratory of MARDI Kluang and rumen fluid was obtained from three castrated bulls in cannulated animal cages in the MARDI Kluang Ruminant Complex (approved by the MARDI Animal Ethics Committee; Approval Number: 20200106/R/MAEC00070). They were fed with their normal diet of 60:40 total mixed rations:Napier silage at 3% of individual body weight which are 424.0 kg, 401.0 kg and 416.0 kg of body weight, respectively. *In vitro* incubation assay was prepared at the digestibility laboratory of MARDI’s Centre of Excellent (Livestock) for a digestibility trial. After discarding the digested sample from the digestibility assay procedures, residue pallets were dried and prepared for NDF distillation and all supernatants were frozen and prepared for short-chain fatty acid determination using gas chromatography (GC) (Agilent Technologies, USA) at Bioassay Laboratory of Livestock Research Centre, MARDI Headquarters, Serdang.

### Experimental Design

In Study 1, 20 units of 200-liter tanks were arranged using the Complete Randomised Design (CRD) in 5 concentration levels of fertiliser with 4 replicates each. All tanks were labelled as C1, C2, C3, C4 and C5 which represent 0.25 g/L, 0.50 g/L, 0.75 g/L, 1.00 g/L and 1.25 g/L, respectively. The arrangement of tanks was done on the 240 m^2^ flat concrete area with 100% sun exposure. By using the same arrangement, this study was repeated four (4) times using different fertilisers: Control (NPK 15:15:15), F1 (broiler manure), F2 (sheep manure) and F3 (cow manure). Each study was conducted for 14 days with daily observation and site maintenance.

For Study 2, twelve units of 200-liter tanks were arranged in 3 treatments with 4 replications each. The tanks were arranged according to CRD and were labelled as T1, T2 and T3 which represent the cultivation of *A. filiculoides* using dried, burned and fresh, respectively. The study was conducted at the same location as Study 1. Area and tank preparation was done on the same day and sampling was done on day-14 after cultivation.

### Preparation of Fertiliser

The livestock manure used in this study was collected from four sources. Behn Meyer Agricare NPK 15:15:15 was bought from the local agriculture store as the Control. The broiler (Fertiliser 1) and sheep manure (Fertiliser 2) were collected from the Poultry Research Complex and Small Ruminant Research Complex at MARDI Serdang, Meanwhile, the cow manure (Fertiliser 3) was collected from the farmer’s cow farm at Sungai Ramal, Kajang, Selangor. The manures were dried using the Force-air circulation oven (Memmert, Germany) at 60°C for 48 h. Next, the dried manure was ground into powder form by using a motorised grain grinder (SIMA, Malaysia). The powdered manure was subsequently weighted using digital balanced (Sartorius Lab Instruments GmbH & Co, Germany) into 50 g, 100 g, 150 g, 200 g and 250 g, respectively. All organic manures were prepared and stored in the dried and cleaned container while the preparation of cultivation tanks was conducted.

### Preparation of *Azolla filiculoides* Culture

The *Azolla filiculoides* were obtained from the local freshwater pond near Sri Serdang Recreation Park were botanically selected according to their botanical morphology and characteristics as described by Saunders and Fowler (1992) and Stafford (2003) and later were cultivated in the HDPE polyethylene water tank with NPK 15:15:15 dilution at the concentration of 0.5 g/L. Cultivation took approximately 4 weeks to produce well mature plant for the experiment initiation culture.

### Harvesting and Yield Determination Test

In both Study 1 and Study 2, all the harvested plant was placed in the plastic strainer to remove excess water from the plant. After sieving under the shaded area for 30 min, the weight of the samples was measured using a digital laboratory balance (Sartorius Lab Instruments GmbH & Co, Germany) and recorded as the wet weight (W^harvest^). There are two main parameters were used to support the biomass of this plant. According to Stewart and Boyd (1999), relative growth rate (RGR) and doubling time (DT) have proved to significantly affect the biomass production of the species. The RGR was described by Jackson (1980) while DT was formulated by Mitchell and Tur (1975). However, due to the purpose of using this parameter as a yield component of *A. filiculoides*, those equations were used based on Astuti and Indriatmoko (2018). The calculation was made to determine the potential of the organic manure and the effect on the growth performance of *A. filiculoides*. The data was calculated using the equation:


$$\text{Relative growth rate},\text{RGR}=\frac{\left[\text{ln }{\text{W}}^{\text{initial}}-\text{ln }{\text{W}}^{\text{harvest}}\right]}{\text{T}}$$

where T = duration of experiment (days); W^initial^ = weight of plant used for initiating the cultivation; and W^harvest^ = weight of fresh plant harvested on ‘T’ time.

The relative growth rate (RGR) of *A. filiculoides* was calculated following the equation:


$$\text{Doubling time},\text{DT}=\frac{\text{ln}2}{\text{RGR}}$$

#### Partial drying and sample preparation

The weight of the container was measured using the top loading balance and all the values had been recorded as W^empty^ to the nearest 0.01 g. The weight of the wet sample was recorded to the nearest 0.01 g as W^wet^. Then, it was dried in the Forced-Air Oven (Binder GmbH, Germany) at 60°C for 72 h. The containers were simultaneously removed and cooled in the desiccator (Merck, Germany) for at least 30 min. Then, the weight of the dried sample was recorded to the nearest 0.01 gram as W^dried^ and was ground using the FRITSCH Cutting Mill PULVERISETTE 15 (Fritsch GmbH, Germany) into 1.0 mm size. The partial dry matter was calculated using this equation:


$$\text{Partial dry matter }(\%)=\frac{{\text{W}}^{\text{dried}}-{\text{W}}^{\text{empty}}}{{\text{W}}^{\text{wet}}}\times 100\%$$

where W^empty^ = weight of empty container in gram; W^wet^ = weight of wet sample with container in gram; W^dried^ = weight of dried sample and container in gram.

### Proximate Analysis

All dried samples were analysed for proximate nutrient composition analysis at the Feed Quality Laboratory MARDI Headquarters. From each replicate, a total of 100 mg samples were taken for the proximate analysis procedure of dry matter (DM), ash, crude protein (CP) and crude fibre (CF) while approximately 400 g of samples were used for crude fat determination procedures (EE). The procedure was according to the method described in the Association of Official Analytical Chemists (AOAC 2005) and Goering (1970).

### Fibre Analysis

Fibre analysis was performed using a detergent distillation procedure developed by Soest (1963). Neutral Detergent Solution (NDS) and Acid Detergent Solution (ADS) were prepared and used for NDF and ADF procedures. Distillation of NDF was conducted using the FibreCap™ system (FOSS, Denmark) while Acid Detergent Fibre (ADF) and Acid Detergent Lignin (ADL) distillation procedures were carried out using a Fibertech™ 8000 system (FOSS, Denmark). Samples that have undergone the ADF analysis process went through the lignin component extraction process using 70% sulfuric acid. Sulfuric acid was drained out slowly and the sample residue was rinsed with distilled water before being dried at 60°C for a period of 24 h. The percentage of NDF, ADF and lignin components were calculated according to Soest (1967).

### Digestibility Analysis

#### Preparation of reagent and chemical

Before rumen fluid collection from the cannulated bulls and subsequent incubations, all necessary reagents and solutions were prepared a few days in advance. To prepare the macromineral solution, 6.2 g potassium dihydrogen phosphate (KH_2_PO_4_), 5.7 g disodium hydrogen phosphate (Na_2_HPO_4_) and 0.6 g magnesium sulfate (MgSO_4_) was dissolved in the 1 L of distilled water. A separate solution containing micronutrients was prepared by dissolving 10.0 g of manganese chloride (MnCl_2_), 13.2 g of calcium chloride (CaCl_2_), 1.0 g of cobalt chloride (COCl_2_) and 8.0 g of ferric chloride (FeCl_3_) in 100 mL of distilled water. The solution was stirred until all salts were fully dissolved. Resazurin (0.1 g) was dissolved in 100 mL of distilled water to create a working solution. On the day of the incubation procedure, 35 g of sodium bicarbonate (NaHCO_3_) and 4 g of ammonium carbonate (NH_4_HCO_3_) were dissolved in 1 L of distilled water to prepare the bicarbonate buffer solution.

### *In vitro* Gas Production Assay

This study employed a modified *in vitro* gas (IVG) assay based on the method described by Blummel and Ørskov (1993). Feed samples (approximately 200 mg) were weighed and placed in 100 mL calibrated glass syringes (Model Fortuna, Germany), following the recommendations of Menke and Steingass (1988). Treatments and replicates were arranged in a Completely Randomised Design (CRD) within a water bath. An anaerobic buffer solution containing essential micro and macronutrients, a reducing agent, and the redox indicator resazurin was added to each syringe along with 10 mL of rumen fluid. Negative control syringes, containing only buffered rumen fluid without any substrate, were also included in triplicate to account for any basal gas production from the rumen fluid itself, as previously described by El-Waziry *et al*. (2016). Cumulative gas production (mL/g dry matter; DM) was measured at regular intervals throughout the incubation period (2, 4, 6, 8, 10, 12, 14, 16, 18, 20, 22, 24, 30, 36, 42, 48, 72 and 96 h) at 39°C, following the protocol established by Zailan *et al*. (2016a). The gas volume produced after 24 h of incubation (GP24) was then used to calculate *in vitro* dry matter digestibility (IVDMD), in vitro organic matter digestibility (IVOMD), and metabolisable energy (ME) using the equations outlined by Menke and Steingass (1988).

### Monitoring Gas Production

Following the addition of the rumen fluid mixture (30 mL) to each syringe, all syringes were placed in the water bath according to their designated treatment groups. Gas production was monitored throughout the incubation at 39°C, with readings taken at regular intervals: 2, 4, 6, 8... up to 96 h. To prevent excessive pressure buildup, gas exceeding 80 mL was carefully released by depressing the syringe plunger until the red marker reached the 30 mL mark. The volume of gas produced was recorded at each reading time point. The gas volume accumulated after 24 h of incubation (GP24), expressed per unit of dry matter (mL/200 mg DM), was then used in the metabolisable energy (ME) calculation equation established by Menke and Steingass (1988).

### Determination of *in vitro* Dry Matter Digestibility (IVDMD) and *in vitro* Organic Matter Digestibility (IVOMD)

Following the 96-hour incubation, the digested residues were carefully transferred to pre-weighed 50 mL centrifuge tubes. Any residual material clinging to the syringes was rinsed out with a pipette during transfer. All tubes were centrifuged simultaneously at 20,000 *x*g for 20 min at 4°C to separate the solid residue pellet from the supernatant liquid. The supernatant was collected for subsequent volatile fatty acid (VFA) analysis. The remaining residue pellets were oven-dried in a Binder ED 23-UL benchtop dryer at 60°C for 24 h. The dried residue weight was recorded for the calculation of IVDMD. The ash content, obtained by combustion at 550°C for 5 h, was used to determine IVOMD. Equations established by Menke *et al*. (1979) and Menke and Steingass (1988) were employed to calculate both IVDMD and IVOMD.

### Statistical Analysis

Means yield, nutrient composition, fibre components and digestibility between treatments were analysed using One Way ANOVA in SPSS version 25 statistical software (IBM Corp. 2017). Significant differences between means were compared using Duncan’s Multiple Range Test (DMRT).

## RESULT AND DISCUSSION

### Impact of Different Types of Fertilisers and Concentrations on *Azolla filiculoides* Growth Performance

**Figure 1:**
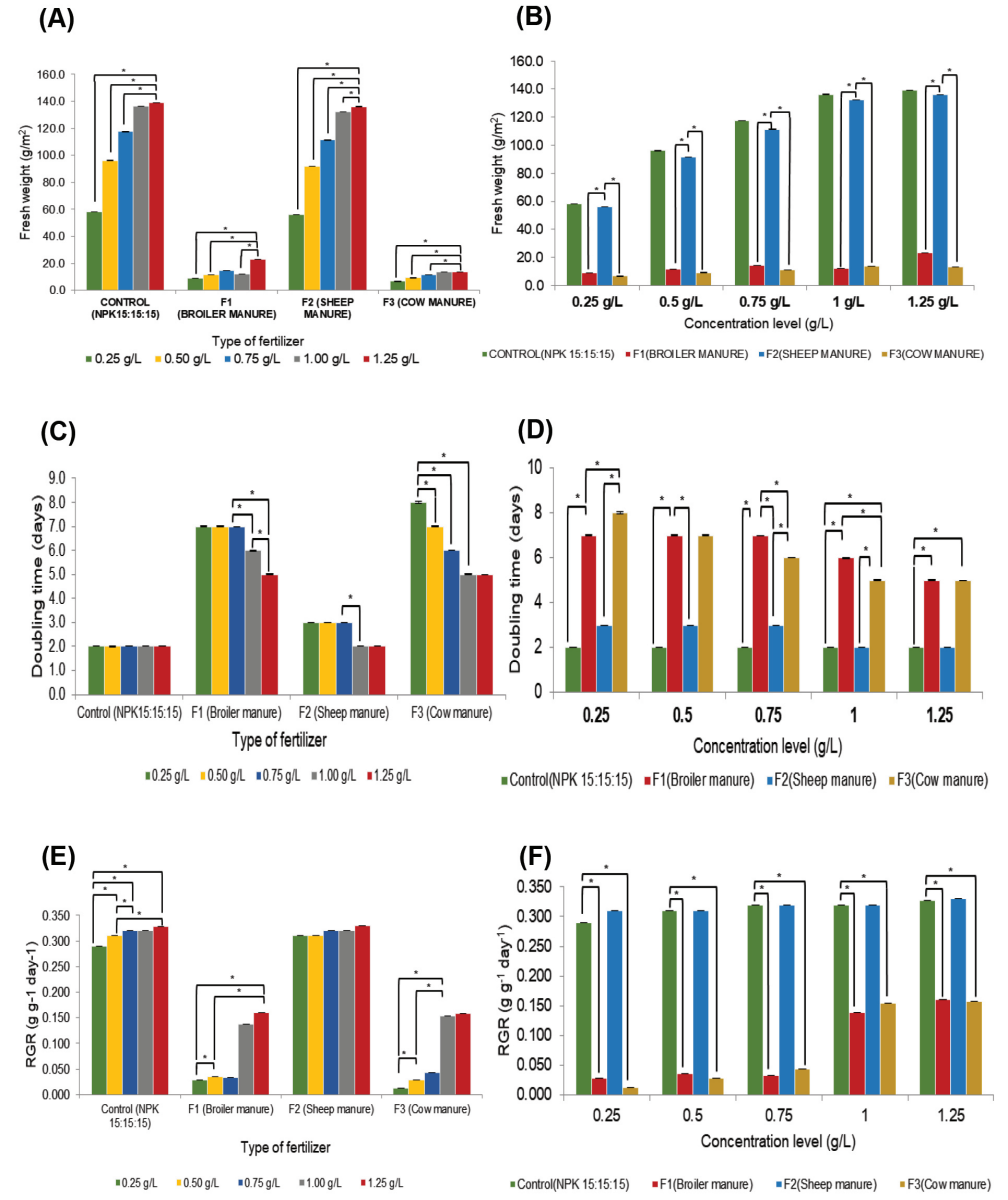
(A–B) Fresh weight; (C–D) doubling time (DT); and (E–F) relative growth rate (RGR) of cultivated *Azolla filiculoides* treated with different fertilisers at different increasing concentrations. (*n* = 4).

Our investigation in Study 1 revealed that cultivating *A. filiculoides* with sheep manure at a concentration of 1.00 g/L yielded the most favourable outcome. This treatment achieved the shortest doubling time (DT) which is in 2 days and produced the highest fresh weight (FW) of 132.2 g/m^2^ and relative growth rate (RGR) of 0.32 g/g/day (Fig. 1). Significantly higher fresh weight (FW) of *A.filiculoides* was achieved using sheep manure (F2) compared to broiler manure(F1) and cattle manure (F3). Notably, FW production with F2 was comparable tothe control treatment using NPK 15:15:15 chemical fertiliser. This suggests that F2could be a cost-effective and sustainable alternative to NPK fertilisers for Azollacultivation on farms. While the optimal concentration of F2 (1.25 g/L) to achieve aFW of 136 g/m^2^ was slightly higher than the control (1.00 g/L), the significantly nocost of sheep manure as this can be obtained freely from sheep farm compared toNPK fertilisers which are chemical-based and require cost makes this differencenegligible for farmers seeking a protein source for their farms, an application of F2could be an alternative way to cultivate this species in more economical in the farms Interestingly, even at a lower concentration (1.0 g/L), F2 produced a significantlyhigher FW (136.1 g/m^2^) compared to F1 (12.2 g/m^2^) and F3 (13.5 g/m^2^). The overall FW range obtained with F2 (56.1 g/m^2^–136.1 g/m^2^) was statistically similar (*p* > 0.05) to the control group’s range (58.0 g/m^2^–139.1 g/m^2^). Slight variations in the range could be attributed to differences in relative growth rate (RGR) and doubling time (DT) observed with different fertiliser types and concentrations.

Sheep manure (F2) emerged as a promising substitute for the conventional NPK 15:15:15 fertiliser for cultivating *A. filiculoides* (Fig. 1). F2 application resulted in a significantly higher relative growth rate (RGR) and shorter doubling time (DT) compared to other fertilisers (F1, F3 and NPK). Notably, F2 at a concentration of 1.0 g/L achieved an optimal RGR of 0.30 g/g/day and a DT of 2 days. This aligns well with the optimal values reported by Da Silva *et al*. (2022) (0.32 g/g/day for RGR and 2.16 days for DT). Furthermore, F2 consistently promoted superior RGR compared to *A. pinnata* (0.15 g/g/day) and *A. caroliana* (0.12 g/g/day) grown using the Hoagland’s nutrient solution (a chemical fertiliser; Kösesakal & Yıldız 2019). This enhanced growth performance with F2 might be attributed to its influence on pH and phosphorus concentration, factors known to be crucial for Azolla growth (Cheng *et al*. 2010; Azab & Soror 2020).

**Figure 2:**
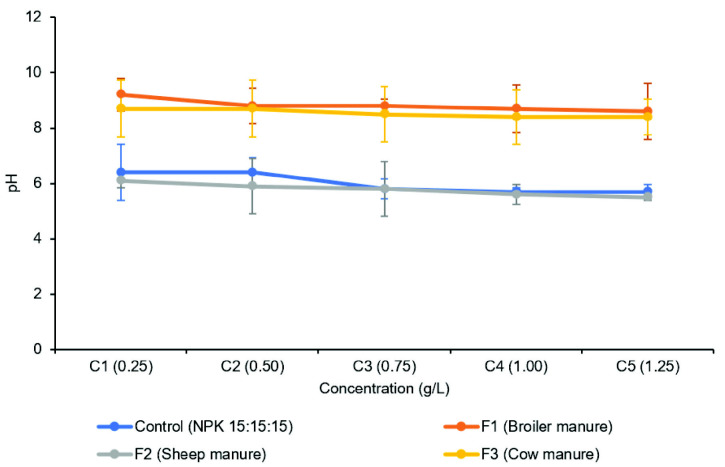
pH value of cultivation media at Day 14.

The pH of the cultivation media throughout the experiment was also monitored and recorded. Interestingly, the pH of media containing sheep manure (F2) was acidic, ranging from pH 5.5 to 6.1, and closer to the pH of the control medium with NPK 15:15:15 fertiliser. In contrast, media containing broiler manure (F1) and cattle manure (F3) exhibited a more alkaline pH. This observation aligns with the findings in Fig. 2, suggesting that *A. filiculoides* thrives better in acidic environments compared to alkaline ones. Fresh weight production is known to be positively correlated with relative growth rate (RGR) and negatively correlated with doubling time (DT). These factors can be influenced by both biotic and abiotic factors. Biotic factors include the cyanobacterial population (*Anabaena azollae*), while abiotic factors include environmental pH and mineral concentration. Notably, F2 treatment, despite having a similar concentration to other fertilisers (F1 and F4), resulted in significantly lower (*p* < 0.05) fresh weight production. This could potentially be attributed to the alkaline nature (pH 8.7–9.2) of the media containing F1 and F4, along with their differing phosphorus content.

Our findings are supported by Azab and Soror (2020), who reported that Azolla growth is optimal in environments with a pH below 7.2. This is further supported by Golzary *et al*. (2020) and Da Silva *et al*. (2022), who identified a pH of 6.5 as the ideal range for Azolla. Additionally, Cheng *et al*. (2010) highlighted the importance of phosphorus for Azolla growth, a notion supported by Williams and Haynes (1995). Their study revealed that sheep manure has a significantly higher phosphorus content (35% P/kg) compared to broiler manure (0.9% P/kg) and cow manure (0.5% P/kg). Handajani (2011) further reinforced this concept by demonstrating that Azolla plants grown in media with a high phosphorus ratio (N:P = 1:3) exhibited increased fresh weight production.

### Impact of Different Types of Fertilisers and Concentrations *on Azolla filiculoides* Nutrient Composition

Study 1 investigated the influence of different fertiliser types and concentrations on the nutrient composition of cultivated *A. filiculoides*. Four fertilisers were examined: NPK 15:15:15 (control), broiler manure (F1), sheep manure (F2) and cow manure (F3). Each fertiliser was applied at five increasing concentrations. The concentration of applied fertiliser significantly affected the dry matter (DM), ash, crude protein (CP), crude fibre (CF) and crude fat (EE) content of *A. filiculoides* (Table 1). Sheep manure (F2) resulted in significantly higher DM and CP compared to broiler manure (F1) and cow manure (F3). At concentrations of 0.75 g/L and 1.00 g/L, F2 produced *A. filiculoides* with DM content (7.0%–7.3%) approaching that of the control. Furthermore, F2 concentrations of C3 and above yielded *A. filiculoides* with CP content ranging from 20.7% to 21.3% DM^−1^. Regardingcrude fibre (CF), essential for ruminant digestion, F1, F2 and F3 achieved optimalconcentrations at C5, C4 and C3, respectively, based on a target crude fibrecontent of 13.4% as reported by Luthfi *et al*. (2018). The higher concentrationsrequired for F1 and F2 compared to the control did not pose a practical challengeas these manure-based fertilisers are readily available on farms.

Ash content, primarily derived from minerals like calcium and phosphorus absorbed by the plant, generally increases with increasing fertiliser concentration. The lowest ash content was observed in *A. filiculoides* grown with the lowest concentration (C1) of F2. In terms of crude fat (EE), all treatments except F3 produced *A. filiculoides* with a crude fat content exceeding 4.0% DM^−1^. The ash content in *A. filiculoides* likely reflects the minerals absorbed during cultivation, categorised as endogenous (e.g., calcium, phosphorus, potassium) or exogenous (minerals not typically found in plants) according to Hoffman (2005). Previous research by Cherryl *et al*. (2014) indicated that Azolla grown using cow manure can produce approximately 2.6% DM^−1^ calcium and 0.3% DM^−1^ phosphorus, suggesting that the ash may be derived from absorbed calcium and phosphorus, classified as endogenous minerals. Additionally, Unadkat and Parikh (2017) reported that aquatic plants exhibit a specific absorption threshold for mineral intake during growth. In this study, the varying fertiliser concentrations significantly affected the ash content in *A. filiculoides*. The lowest concentration (C1) of F2 resulted in the lowest ash content and the highest organic matter content, potentially making it more favourable as an animal feed. The crude fibre (CF) content of *A. filiculoides* plays a critical role in the digestion and rumination process of animals. As expected, CF content increased with increasing fertiliser concentration. However, *A. filiculoides* grown with F2 at concentrations C3 to C5 exhibited CF content closest to the optimal level (13.4% DM^−1^) for ruminants as reported by Luthfi *et al*. (2018).

### Impact of Different Types of Fertilisers and Concentrations *on Azolla filiculoides* Fibre Component

Table 2 presents the effects of different fertiliser types and increasing concentrations on the fibre component composition of *Azolla filiculoides*. The table details the content of neutral detergent fibre (NDF), acid detergent fibre (ADF) and lignin within the cultivated Azolla. The structure and composition of fibre components in a food is a major factor that affects the level of digestion and absorption of nutrients in the animal system. Furthermore, it is one of the important components in the diet of livestock, especially ruminants. In this study, higher concentration media for *A*. *filiculoides* cultivation led to an increment in the composition of NDF. Neutral detergent fibre (NDF) is a major cell wall component in plants and directly affects forage digestibility by ruminants. Lower NDF values generally translate to easier breakdown during rumination, potentially leading to increased voluntary feed intake. Therefore, NDF content is a crucial factor considered by nutritionists and farmers for optimising dry matter intake (DMI) in ruminant animals. Our study investigated the influence of fertiliser type and concentration on NDF content in cultivated *A. filiculoides*. As shown in Table 2, increasing fertiliser concentration generally resulted in elevated NDF values for Azolla grown with NPK 15:15:15 (control), broiler manure (F2), sheep manure (F3) and cow manure (F4) across all concentrations (0.25 g/L to 1.25 g/L). Azolla cultivated with NPK fertiliser (F1) exhibited NDF values ranging from 34.4% to 37.6% DM^−1^. Interestingly, cow manure (F4) at the lowest concentrations (0.25 g/L and 0.50 g/L) resulted in significantly lower (*p* < 0.05) NDF content compared to other treatments. Conversely, using sheep manure (F3) at concentrations exceeding 0.75 g/L significantly decreased the NDF content in *A. filiculoides*.

Acid detergent fibre (ADF) represents the fraction of forage or feed high in lignin and cellulose, which influences digestibility by ruminants. Generally, lower ADF content translates to higher digestibility, potentially improving rumen activity and dry matter intake (DMI). Conversely, forages with high ADF content typically have lower energy availability. In this study, *A. filiculoides* grown with NPK fertiliser (F1) exhibited an ADF range of 28.9%–29.9% DM^−1^. Interestingly, all manure-based fertilisers (broiler manure (F2), sheep manure (F3) and cow manure (F4)) resulted in significantly lower ADF content (23.2%–29.7% DM^−1^) compared to NPK. Notably, the minimum ADF values for Azolla grown with F2, F3 and F4 were 23.9%, 24.6% and 23.2% DM^−1^, respectively. Additionally, sheep manure (F3) at concentrations of 0.25 g/L and 0.50 g/L produced Azolla with significantly lower (*p* < 0.05) ADF content compared to NPK, broiler manure and cow manure. Importantly, no significant differences in ADF content were observed at higher fertiliser concentrations across all treatments. The acid detergent lignin (ADL) procedure removes soluble components like hemicellulose and minerals from feed or forage, leaving behind indigestible lignin and some residual protein in the cell wall. This study revealed a positive correlation between lignin content and manure concentration. The percentage of lignin in Azolla grown with NPK (F1) ranged from 4.5% to 9.2% DM^−1^, which was significantly higher than the ADL of Azolla grown with sheep manure (F3) and cow manure (F4) at 1.00 g/L (C4). Our findings indicate that specific fertiliser types and concentrations can influence lignin levels in Azolla. The highest lignin content (8.7%–9.5% DM^−1^) was observed in Azolla grown with NPK fertiliser (F1) at 1.00 g/L (C4) compared to other fertilisers. Interestingly, across all concentrations, the highest lignin content (9.5% DM^−1^) was measured in Azolla grown with sheep manure (F2) at 1.25 g/L (C5), with no significant differences observed between fertiliser types at this concentration.

### Impact of Different Manure Preparation Procedures on *Azolla filiculoides* Growth Performance

**Figure 3:**
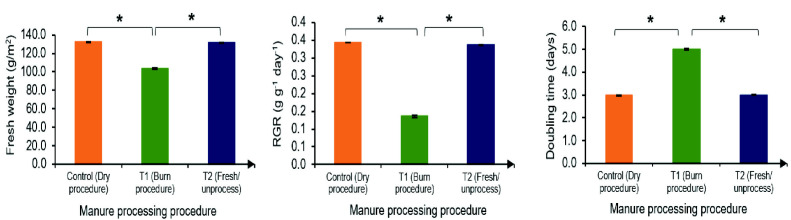
Fresh weight, relative growth rate and doubling time of cultivated *Azolla filiculoides* using fertiliser with different preparation procedure.

Findings from Study 1 were applied in Study 2. Sheep manure (F2) was used as a fertiliser to cultivate *Azolla filiculoides*. Fig. 3 shows the influence of different manure preparation procedures (T1: drying, T2: burning, T3: fresh) on fresh weight, doubling time (DT) and relative growth rate (RGR) of cultivated *A. filiculoides*. Sheep manure used in this study resulted in fresh weight production ranging from 103.6 g/m^2^ to 132.2 g/m^2^ (as shown in the image), with DT and RGR values between 3 and 5 days and 0.136 to 0.343 g/g/day, respectively. Manure prepared using procedures T1 (drying) and T3 (fresh) yielded significantly higher (*p* < 0.05) fresh weight compared to T2 (burning). Importantly, T1 served as the control treatment in this experiment, while T2 and T3 were chosen for their practicality and economic benefits, as they do not require drying facilities. The T3 procedure (fresh manure) resulted in the highest fresh weight (131.4 g/m^2^), exceeding the 55.9 g/m^2^ reported by Azab and Soror (2020) who applied weekly doses of 1.0 kg broiler manure. In their study, broiler manure fertiliser was found to enhance growth performance and protein content in *A. filiculoides* compared to chemical fertilisers. The superior fresh weight production observed with T3 manure can likely be attributed to the shorter DT and higher RGR values achieved with this treatment. Azab and Soror (2020) reported a doubling time of within 7 days and an RGR of 0.4 g/g/day for most Azolla species. Our findings indicate that a shorter DT (3 days) and an RGR of 0.34 g/g/day are achievable for *A. filiculoides* propagation using fertiliser prepared with the T3 procedure. These results are consistent with those of Golzary *et al*. (2020), who found that *A. filiculoides* grown under controlled conditions (22°C, 20lx light intensity, 75% humidity, pH 6.4) achieved a doubling time of 2.1 days.

### Impact of Different Manure Preparation Procedures on *Azolla filiculoides* Nutrient Composition

**Figure 4:**
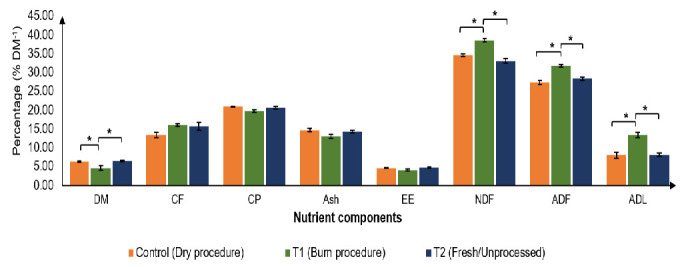
Nutrient composition of cultivated *Azolla filiculoides* using a fertiliser with different preparation procedures (*n* = 4).

Fig. 4 shows the effect of different manure preparation procedures (T1: drying, T2: burning, T3: fresh) on the nutrient composition of cultivated *A. filiculoides*. Although treatment T2 resulted in lower dry matter (DM) content compared to T1 and T3 (as shown in the image), there were no significant differences (*p* > 0.05) in ash, crude protein (CP), crude fat (EE) and crude fibre (CF) content across all treatments (represented by the vertical bars in the image). Azolla grown with sheep manure prepared using any of these procedures yielded CP content ranging from 19.79% to 20.93% DM^−1^, CF content between 13.02% and 14.66% DM^−1^, and ash content from 13.39% to 16.03% DM^−1^. Our study suggests that the essential nutrient composition (CP, CF, ash) of *A. filiculoides* was lower compared to the values reported for *A. pinnata* grown with cow manure and Superphosphate fertiliser (Anitha *et al*.2016; Khursheed *et al*. 2019). However, *A. filiculoides* demonstrates potential aslivestock feed based on its higher CP content compared to Napier Zanzibar grass(19.4% DM^−1^; Haryani *et al*. 2018) and lower CF content than Napier Indian grass(29.60% DM^−1^; Haryani *et al*. 2018). Furthermore, the DM content of *A. filiculoides*(below 10%) makes it more suitable as a dietary supplement for livestock compared to Napier grass species, which typically have a DM content exceeding 10% (Zailan*et al*. 2016a).

Fig. 4 also presents the fibre component composition (NDF, ADF, ADL) of *A. iliculoides* cultivated using sheep manure processed with different methods: drying (T1), burning (T2) and unprocessed/fresh (T3). Azolla grown with manure prepared via drying (T1) exhibited the lowest percentages of NDF (34.56% DM^−1^), ADF (27.30% DM^−1^) and ADL (8.00% DM^−1^), as shown in the image. In contrast, burning manure (T2) resulted in significantly higher (*p* < 0.05) NDF, ADF and ADL content compared to T1. Interestingly, Azolla cultivated with manure prepared using the fresh treatment (T3) did not differ significantly (*p* > 0.05) from that grown with dried manure (T1). The NDF content of Azolla produced with fresh manure (T3; 33.02% DM^−1^) was lower than that reported for *Brachiaria humidicola* and *Pennisetum purpureum* at maturity (120 and 90 days, respectively (Mupenzi *et al*. 2017). Additionally, the NDF and ADL values of T3 samples were comparable to those of Napier Dwarf grass at 4 weeks–6 weeks of age (Zailan *et al*. 2016a). These findings suggest that *A. filiculoides* cultivated with fresh manure has the potential for easier degradation during rumination, potentially leading to increased feed intake and palatability. While the ADL content of Azolla was higher than that reported for Napier Dwarf grass at 8 weeks (7.14% DM^−1^; Zailan *et al*. 2016b), it remains relatively low. According to Liu *et al*. (2021), a decrease in plant nitrogen uptake can lead to increased lignin, cellulose, and hemicellulose content in plant cells. This phenomenon might explain the higher fibre content observed in Azolla grown with burned manure (T2), potentially due to the loss of nitrogen from ammonia during combustion compared to drying or using fresh manure.

### Impact of Different Sheep Manure Preparation Procedures on *Azolla filiculoides* Gas Production and Digestibility

**Figure 5:**
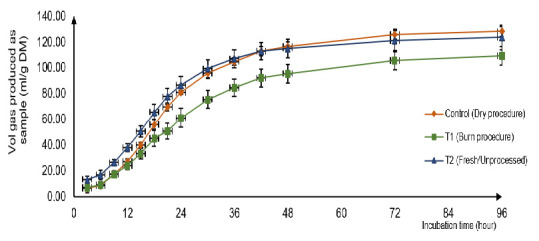
96-hours *in vitro* gas production of cultivated *Azolla filiculoides* using fertiliser with different preparation procedure.

The effect of different preparation procedures of sheep manure as fertiliser for *Azolla filiculoides* cultivation on Azolla’s accumulated *in vitro* gas production is shown in Fig. 5. The way fibre components are arranged and structured within plant cells plays a major role in forage digestibility. Analysis of these components, including neutral detergent fibre (NDF), acid detergent fibre (ADF) and lignin, provides valuable insights into how easily complex sugars can be broken down into simpler forms by rumen microbes. Fig. 5 depicts the effect of different sheep manure preparation procedures (T1: drying, T2: burning, T3: fresh) on cumulative gas production. *In vitro* gas production serves as a proxy for rumen microbial activity associated with nutrient breakdown, digestion and absorption over a 24-h to 48-h period. Sheep manure prepared using the T3 procedure (freshmanure) resulted in the highest cumulative gas production (between 86.85 mL/DM and 115.11 mL/DM) at both 24 h and 48 h of incubation, as shown in thegraph. These values were significantly higher (*p* < 0.05) compared to thoseobserved with T2 (burning; 61.27 mL/DM–95.61 mL/DM). Our findings indicatethat unprocessed sheep manure (T3) promotes greater cumulative gas volume (*p* < 0.05) during the digestion of *A. filiculoides* within 48 h. In contrast, manure prepared via burning (T2) produced the lowest cumulative gas volume (*p* < 0.05) compared to T1 and T3. This difference can likely be attributed to the susceptibility of the manure to breakdown by rumen microorganisms during rumination. *A. filiculoides* cultivated with unprocessed manure (T3) also exhibited significantly higher (*p* < 0.05) cumulative gas production at 24 h compared to T2. No significant differences in gas production were observed among treatments between 48 h and 96 h of incubation. The cumulative gas volumes at 96 h were 128.04 mL/DM, 109.27 mL/DM, and 123.47 mL/DM for T1, T2, and T3, respectively. These results collectively suggest that fresh sheep manure (T3) enhances the digestibility of *A. filiculoides* by rumen microorganisms. This may be due to a more favourablecomposition or structure of the manure that facilitates microbial breakdown.

**Figure 6:**
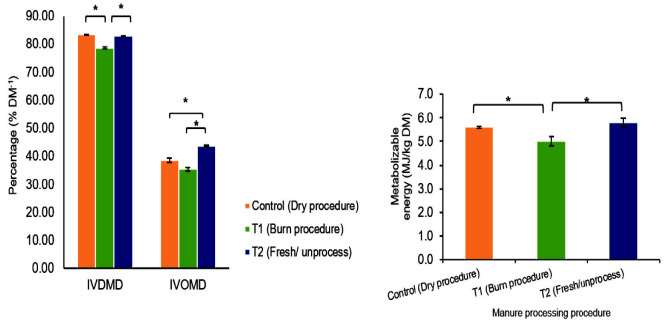
Accumulated *in vitro* gas production (GP), *in vitro* dry matter digestibility (IVDMD), *in vitro* organic matter digestibility (IVOMD), and metabolisable energy (ME) of cultivated *Azolla filiculoides* using sheep manure with different preparation procedures.

In ruminant nutrition, *in vitro* digestibility parameters like IVDMD and IVOMD are crucial for assessing nutrient absorption. IVOMD specifically reflects the portion of organic matter directly digested by the ruminant digestive system. It is commonly used as an indicator of energy availability and can potentially predict microbial protein synthesis within the rumen. While organic manure preparation procedures can influence the formation and structure of plant cell wall components, they can also affect the overall digestibility of the plant as a ruminant feed source. Fig. 6 explores the effect of different manure preparation methods (T1: drying, T2: burning, T3: fresh) on IVDMD, IVOMD and metabolisable energy (ME) of cultivated *A. filiculoides*. Our study revealed that IVDMD values were significantly higher(*p* < 0.05) in Azolla samples grown with unprocessed manure (T3) and driedmanure (T1) compared to those grown with burned manure (T2) (as shown inthe image). These differences can be attributed to the composition and formationof NDF, ADF and acid detergent lignin (ADL) within the plant cells. The burningprocess in T2 disrupts covalent bonds between nitrogen (N) and hydrogen (H)elements in ammonia (NH_3_) present in sheep manure. This loss of nitrogen leadsto a higher carbon-to-nitrogen (C:N) ratio, which consequently promotes theformation of cellulose and lignin in plant cells (Liu *et al*. 2021). Increased celluloseand lignin content within the cells directly translates to a longer catabolism process, ultimately reducing the rate of nutrient degradation in the rumen (Mertens & Grant 2020). Our findings also demonstrate that different manure preparation proceduressignificantly affect the IVOMD of *A. filiculoides*. Azolla cultivated with fresh manure(T3) exhibited the highest IVOMD level (43.7%), followed by T1 (38.7%) and T2(35.4%). These results suggest that fresh manure (T3) promotes greater organicmatter digestibility compared to other preparation methods. Furthermore, theburning procedure (T2) resulted in significantly lower (*p* < 0.05) ME levels compared to T1 and T3. The IVOMD, IVDMD and ME values observed in this study werehigher than those reported for wild-grown *Azolla pinnata* by Parashuramulu *et al*.2013. However, these values were lower than those measured for four Napiergrass species, which ranged from 7.28 to 8.76 MJ/kg DM (Zailan *et al*. 2016a).

## CONCLUSION

This study investigated how fertiliser type, processing method and concentration impact *Azolla filiculoides* growth, nutrient composition and digestibility. Our findings recommend fresh sheep manure (T3 procedure) at a concentration of 1.00 g/L as the most practical and economical fertiliser for Azolla cultivation. This approach achieves high yield (132.2 g/m^2^ fresh weight), promotes rapid growth (shorter doubling time, higher relative growth rate), and provides adequate protein (20.7%–21.3%) and fibre (14.45%–15.7%) content for ruminant diets. Additionally, the fibre profile (NDF, ADF) resembles Napier grass, enhancing palatability and digestibility. Furthermore, Azolla grown with T3 manure exhibits superior digestibility (high IVDMD, IVOMD, and ME) compared to other methods. Importantly, this method offers the potential for significant annual yield (31.2 MT/ha) without fertiliser cost. In conclusion, fresh sheep manure (T3) at 1.00 g/L presents a cost-effective and practical solution for cultivating *Azolla filiculoides* as a valuable source of fibre and protein for livestock feed.

## Figures and Tables

**Table 1:** Dry matter, ash, crude protein, crude fibre and crude fat composition of cultivated *Azolla filiculoides* in different concentrations.

Parameter	Manure concentration (Means ± S.E.M)				

	C1(0.25 g/L)	C2(0.50 g/L)	C3(0.75 g/L)	C4(1.00 g/L)	C5(1.25 g/L)
Dry matter (%)					

Control: NPK 15:1515	8.5 ± 0.17^a^, ^x^	7.4 ± 0.08^b^, ^x^	7.4 ± 0.35^b^, ^x^	7.5 ± 0.11^b^, ^x^	7.6 ± 0.08^b^, ^w^
F1: Broiler manure	7.8 ± 1.02^a^, ^xy^	6.7 ± 0.16^ab^, ^xy^	5.8 ± 0.33^bc^, ^y^	5.5 ± 0.65^bc^, ^y^	4.1 ± 0.17^c^, ^z^
F2: Sheep manure	6.4 ± 0.14^b^, ^yz^	6.2 ± 0.14^b^, ^y^	7.0 ± 0.09^a^, ^x^	7.3 ± 0.23^a^, ^x^	6.9 ± 0.18^a^, ^x^
F3: Cow manure	5.6 ± 0.64^a^, ^z^	6.6 ± 0.48^a^, ^y^	5.9 ± 0.24^a^, ^y^	5.7 ± 0.73^a^, ^y^	5.5 ± 0.15^a^, ^y^

Ash (% DM^−1^)					

Control: NPK 15:1515	17.6 ± 0.19^c^, ^x^	19.7 ± 0.17^a^, ^x^	19.6 ± 0.13^ab^, ^x^	19.6 ± 0.16^ab^, ^x^	19.2 ± 0.07^b^, ^x^
F1: Broiler manure	17.6 ± 0.13^c^, ^x^	18.4 ± 0.12^b^, ^y^	19.6 ± 0.19^a^, ^x^	19.5 ± 0.17^a^, ^x^	18.3 ± 0.10^b^, ^y^
F2: Sheep manure	13.6 ± 0.14^e^, ^y^	17.6 ± 0.15^d^, ^z^	18.5 ± 0.10^c^, ^y^	19.6 ± 0.17^a^, ^x^	19.2 ± 0.06^b^, ^x^
F3: Cow manure	17.6 ± 0.06^b^, ^x^	18.6 ± 0.13^a^, ^y^	18.7 ± 0.06^a^, ^y^	17.5 ± 0.17^b^, ^y^	17.4 ± 0.18^b^, ^z^

Crude protein (% DM^−1^)					

Control: NPK 15:1515	18.2 ± 0.15^d^, ^x^	20.0 ± 0.03^c^, ^yx^	21.1 ± 0.26^b^, ^x^	23.4 ± 0.57^a^, ^x^	23.6 ± 0.30^a^, ^x^
F1: Broiler manure	17.9 ± 0.83^c^, ^x^	18.6 ± 0.57^c^, ^x^	19.5 ± 0.44^bc^, ^y^	20.2 ± 0.03^ab^, ^yz^	21.5 ± 0.36^a^, ^y^
F2: Sheep manure	17.3 ± 0.65^c^, ^x^	19.2 ± 0.49^b^, ^x^	20.7 ± 0.21^a^, ^x^	21.2 ± 0.19^a^, ^y^	21.3 ± 0.18^a^, ^y^
F3: Cow manure	14.7 ± 0.56^c^, ^y^	16.7 ± 0.68^b^, ^y^	19.0 ± 0.31^a^, ^y^	19.4 ± 0.39^a^, ^z^	20.2 ± 0.30^a^, ^z^

Crude fibre (% DM^−1^)					

Control: NPK 15:1515	10.4 ± 0.05^d^, ^x^	11.3 ± 0.11^c^, ^y^	14.4 ± 0.13^b^, ^x^	14.6 ± 0.16^b^, ^y^	16.6 ± 0.14^a^, ^x^
F1: Broiler manure	8.5 ± 0.14^e^, ^z^	9.7 ± 0.11^d^, ^z^	11.5 ± 0.13^c^, ^z^	12.5 ± 0.07^b^, ^z^	16.2 ± 0.07^a^, ^y^
F2: Sheep manure	10.4 ± 0.16^e^, ^x^	11.5 ± 0.17^d^, ^y^	12.3 ± 0.23^c^, ^y^	14.4 ± 0.15^b^, ^y^	15.7 ± 0.11^a^, ^z^
F3: Cow manure	9.5 ± 0.17^e^, ^y^	12.5 ± 0.20^d^, ^x^	14.6 ± 0.09^c^, ^x^	16.4 ± 0.17^b^, ^x^	17.3 ± 0.12^a^, ^w^

Crude fat (% DM^−1^)					
Control: NPK 15:1515	4.2 ± 0.04^b^, ^x^	4.4 ± 0.18^b^, ^x^	4.5 ± 0.14^b^, ^x^	4.5 ± 0.09^b^, ^x^	4.9 ± 0.04^a^, ^x^
F1: Broiler manure	4.2 ± 0.17^a^, ^x^	4.3 ± 0.20^a^, ^x^	4.2 ± 0.13^a^, ^xy^	4.3 ± 0.12^a^, ^x^	4.5 ± 0.14^a^, ^y^
F2: Sheep manure	4.1 ± 0.06^c^, ^x^	4.1 ± 0.05^c^, ^x^	4.1 ± 0.00^bc^, ^y^	4.3 ± 0.08^ab^, ^x^	4.4 ± 0.06^a^, ^y^
F3: Cow manure	3.4 ± 0.14^a^, ^y^	3.5 ± 0.14^a^, ^y^	3.7 ± 0.16^a^, ^z^	3.7 ± 0.10^a^, ^y^	3.7 ± 0.19^a^, ^z^

*Note*:

a,b,c,d Different superscript letters within the row show significant differences between treatments as determined by Duncan’s post-hoc multiple comparisons (p < 0.05).

x,y,z Different superscript letters within the column show significant differences in treatment as determined by Duncan’s post-hoc multiple comparisons (p < 0.05). n = 4.

**Table 2:** Fibre components of cultivated *Azolla filiculoides in* different concentrations.

Fertiliser	Manure concentration (Means ± S.E.M)				

	C1(0.25 g/L)	C2(0.50 g/L)	C3(0.75 g/L)	C4(1.00 g/L)	C5(1.25 g/L)
Neutral detergent fibre (% DM^−1^)					

Control: NPK 15:1515	34.4 ± 0.14^d^, ^x^	35.5 ± 0.15^c^, ^x^	36.3 ± 0.08^b^, ^x^	37.4 ± 0.15^a^, ^x^	37.6 ± 0.19^a^, ^x^
F1: Broiler manure	34.4 ± 0.08^c^, ^x^	35.1 ± 0.03^b^, ^x^	36.5 ± 0.12^a^, ^x^	36.5 ± 0.22^a^, ^y^	36.5 ± 0.17^a^, ^y^
F2: Sheep manure	33.4 ± 0.14^c^, ^y^	33.5 ± 0.18^c^, ^z^	35.5 ± 0.13^b^, ^y^	36.7 ± 0.15^a^, ^y^	36.6 ± 0.08^a^, ^y^
F3: Cow manure	33.5 ± 0.16^d^, ^y^	34.7 ± 0.10^c^, ^y^	36.4 ± 0.18^b^, ^x^	37.6 ± 0.16^a^, ^x^	36.6 ± 0.12^b^, ^y^

Acid detergent fibre (% DM^−1^)					

Control: NPK 15:1515	28.9± 0.29^a^, ^y^	29.7 ± 0.43^a^, ^y^	29.4 ± 0.52^a^, ^y^	29.2 ± 0.47^a^, ^z^	29.9 ± 0.36^a^, ^z^
F1: Broiler manure	23.9 ± 0.70^b^, ^z^	29.6 ± 0.06^a^, ^y^	29.7 ± 0.89^a^, ^y^	29.0 ± 0.59^a^, ^z^	29.1 ± 0.55^a^, ^z^
F2: Sheep manure	24.6 ± 0.24^c^, ^z^	25.2 ± 0.26^c^, ^z^	27.1 ± 0.27^b^, ^z^	27.6 ± 0.57^b^, ^z^	28.8 ± 0.09^a^, ^z^
F3: Cow manure	23.2 ± 1.79^b^, ^z^	25.9 ± 2.03^ab^, ^z^	26.2 ± 0.23^ab^, ^z^	28.0 ± 0.13^a^, ^z^	28.8 ± 0.47^a^, ^z^

Acid detergent lignin (% DM^−1^)					

Control: NPK 15:1515	4.5 ± 0.17^c^, ^y^	6.0 ± 0.19^b^, ^z^	8.7 ± 0.37^a^, ^y^	9.0 ± 0.06^a^, ^x^	9.2 ± 0.08^a^, ^z^
F1: Broiler manure	3.4 ± 0.17^d^, ^z^	7.7 ± 0.22^c^, ^x^	8.1 ± 0.36^bc^, ^yz^	8.6 ± 0.17^b^, ^xy^	9.5 ± 0.25^a^, ^z^
F2: Sheep manure	4.1 ± 0.30^d^, ^y^	5.6 ± 0.07^c^, ^z^	7.4 ± 0.21^b^, ^z^	7.6 ± 0.48^b^, ^z^	8.9 ± 0.20^a^, ^z^
F3: Cow manure	4.2 ± 0.15^d^, ^y^	6.7 ± 0.36^c^, ^y^	7.3 ± 0.41^bc^, ^z^	8.0 ± 0.02^b^, ^yz^	8.9 ± 0.11^a^, ^z^

*Note*:

a,b,c,d Different superscript letters within the row show significant differences between treatments as determined by Duncan’s post-hoc multiple comparisons (*p* < 0.05).

x,y,z Different superscript letters within the column show significant differences in treatment as determined by Duncan’s post-hoc multiple comparisons( *p* < 0.05). *n* = 4.

## References

[b1-tlsr_35-3-265] Acharya P, Mohanty GP, Pradhan CR, Mishra SK, Beura NC, Moharana B (2015). Exploring the effects of inclusion of dietary fresh Azolla on the performance of White Pekin broiler ducks. Veterinary World.

[b2-tlsr_35-3-265] Alhrout HH, Akash MW, Hajazin RK (2018). Effect of farm yard manure and NPK on the yield and some growth components of tomato (*Lycopersicum esculentum*). Research on Crops.

[b3-tlsr_35-3-265] Anitha KC, Rajeshwari YB, Prasanna SB, Shree JS (2016). Nutritive evaluation of Azolla as livestock feed. Journal of Experimental Biology and Agricultural Sciences.

[b4-tlsr_35-3-265] Ansal MD (2020). Azolla for socio-economic development of farming community and environmental benefits. Journal of Krishi Vigyan.

[b5-tlsr_35-3-265] Arora A, Saxena S (2005). Cultivation of *Azolla microphylla* biomass on secondary-treated Delhi municipal effluents. Biomass and Bioenergy.

[b6-tlsr_35-3-265] Association of Official Analytical Chemists (AOAC) (2005). Official method of Analysis.

[b7-tlsr_35-3-265] Astuti LP, Indriatmoko I (2018). Ability aquatic plants to reduce organic matters and phosphate pollution for improve water quality. Jurnal Teknologi Lingkungan.

[b8-tlsr_35-3-265] Azab E, Soror AFS (2020). Physiological behaviour of the aquatic plant Azolla sp. in response to organic and inorganic fertilizers. Plants.

[b9-tlsr_35-3-265] Bhatt N, Singh NP, Singh AK, Kandpal D, Chaudhary P, Patoliya P (2020). Azolla: A potent unconventional feed and its effect of feeding on various livestock species – A review. Journal of Entomology and Zoology Studies.

[b10-tlsr_35-3-265] Bhujel A, Rizal G (2022). Exploring use of Azolla as the potential livestock feed resources: A review. Bhutan Journal of Animal Science.

[b11-tlsr_35-3-265] Blummel M, Ørskov ER (1993). Comparison of in vitro gas production and nylon-bag degradability of roughage in predicting feed intake in cattle. Animal Feed Science and Technology.

[b12-tlsr_35-3-265] Cary PR, Weerts PG (1992). Growth and nutrient composition of *Azolla pinnata* R. Brown and *Azolla filiculoides* Lamarck as affected by water temperature, nitrogen and phosphorus supply, light intensity, and pH. Aquatic Botany.

[b13-tlsr_35-3-265] Cheng W, Sakai H, Matsushima M, Yagi K, Hasegawa T (2010). Response of the floating aquatic fern *Azolla filiculoides* to elevated CO2, temperature, and phosphorus levels. Hydrobiologia.

[b14-tlsr_35-3-265] Cherryl DM, Prasad RMV, Jagadeeswara Rao S, Jayalaxmi P, Srinivas Kumar D (2014). A study on the nutritive value of *Azolla pinnata.*. Livestock Research International.

[b15-tlsr_35-3-265] Da Silva MEJ, Mathe LOJ, Van Rooyen IL, Brink HG, Nicol W (2022). Optimal growth conditions for *Azolla pinnata* R. Brown: Impacts of light intensity, nitrogen addition, pH control, and humidity. Plants.

[b16-tlsr_35-3-265] Dail HW, He Z, Erich MS, Honeycutt CW (2007). Effect of drying on phosphorus distribution in poultry manure. Communications in Soil Science and Plant Analysis.

[b17-tlsr_35-3-265] De Wet LPD, Schoonbee HJ, Pretorius J, Bezuidenhout LM (1990). Bioaccumulation of selected heavy metals by the water fern, *Azolla filiculoides* Lam. in a wetland ecosystem affected by sewage, mine and industrial pollution. Water SA.

[b18-tlsr_35-3-265] Elghandour MM, Reddy PRK, Salem AZ, Reddy PPR, Hyder I, Barbabosa-Pliego A, Yasaswini D (2018). Plant bioactives and extracts as feed additives in horse nutrition. Journal of Equine Veterinary Science.

[b19-tlsr_35-3-265] El-Sobky ESE (2017). Effect of burned rice straw, phosphorus and nitrogen fertilization on wheat (*Triticum aestivum* L.). Annals of Agricultural Sciences.

[b20-tlsr_35-3-265] El-Waziry A, Alkoaik F, Khalil A, Metwally H, Fulleros R (2016). Nutrient components and in vitro digestibility of treated and untreated date palm wastes with mushroom (*Pleurotus florida*). Advances in Animal and Veterinary Sciences.

[b21-tlsr_35-3-265] Goering HK, Goering HK, Van Soest PJ (1970). Forage fiber analyses (apparatus, reagents, procedures, and some applications). Agriculture Handbook.

[b22-tlsr_35-3-265] Golzary A, Hosseini A, Saber M (2020). *Azolla filiculoides* as a feedstock for biofuel production: Cultivation condition optimization. International Journal of Energy and Water Resources.

[b23-tlsr_35-3-265] Handajani H (2011). Optimation of nitrogen and phosphorus in Azolla growth as biofertilizer. Makara Journal of Technology.

[b24-tlsr_35-3-265] Haryani H, Norlindawati AP, Norfadzrin F, Aswanimiyuni A, Azman A (2018). Yield and nutritive values of six Napier (*Pennisetum purpureum*) cultivars at different cutting age. Malaysian Journal of Veterinary Research.

[b25-tlsr_35-3-265] Hoffman PC (2005). Ash content of forages. Focus on Forage.

[b26-tlsr_35-3-265] Huggins D (2007). Evaluation of Azolla plant as an alternative stock feed source.

[b27-tlsr_35-3-265] IBM Corp (2017). IBM SPSS Statistics for Windows.

[b28-tlsr_35-3-265] Jackson GA (1980). Phytoplankton growth and zooplankton grazing in oligotrophic oceans. Nature.

[b29-tlsr_35-3-265] Khursheed I, Masud S, Khan A, Khan N, Kour S, Dua S, Khursheed I (2019). Proximate evaluation of Azolla pinnata as sustainable feed supplement for poultry. Journal of Pharmacognosy and Phytochemistry.

[b30-tlsr_35-3-265] Kollah B, Patra AK, Mohanty SR (2016). Aquatic microphylla Azolla: A perspective paradigm for sustainable agriculture, environment and global climate change. Environmental Science and Pollution Research.

[b31-tlsr_35-3-265] Kösesakal T, Yıldız M (2019). Growth performance and biochemical profile of *Azolla pinnata* and *Azolla caroliniana* grown under greenhouse conditions. Archives of Biological Sciences.

[b32-tlsr_35-3-265] Krishna MM, Seshaiah CV, Anitha A, Srinivas D (2022). Wastewater treatment from dairy farm by using Azolla (*Azolla pinnata*). The Pharma Innovation Journal.

[b33-tlsr_35-3-265] Liu XM, Gu WR, Li CF, Jing LI, Shi WEI (2021). Effects of nitrogen fertilizer and chemical regulation on spring maize lodging characteristics, grain filling and yield formation under high planting density in Heilongjiang Province, China. Journal of Integrative Agriculture.

[b34-tlsr_35-3-265] Luthfi N, Restitrisnani V, Umar M (2018). The optimation of crude fiber content of diet for fattening madura beef cattle to achieve good A:P ratio and low methane production. IOP Conference Series: Earth and Environmental Science.

[b35-tlsr_35-3-265] Majumdar J, Rajagopal V, Shantaram MV (1993). Rock phosphate is an effective P carrier for Azolla. International Rice Research Notes.

[b36-tlsr_35-3-265] Menke KH, Steingass H (1988). Estimation of the energetic feed value obtained from chemical analysis and in vitro gas production using rumen fluid. Animal Research and Development.

[b37-tlsr_35-3-265] Menke KH, Raab L, Salewski A, Steingass H, Fritz D, Schneider W (1979). The estimation of the digestibility and metabolizable energy content of ruminant feeding stuffs from the gas production when they are incubated with rumen liquor in vitro. The Journal of Agricultural Science.

[b38-tlsr_35-3-265] Mertens DR, Grant RJ (2020). Digestibility and intake. Forages: The Science of Grassland Agriculture.

[b39-tlsr_35-3-265] Mézes M (2018). Alternative protein sources in the nutrition of farm animals. Acta Agraria Debreceniensis.

[b40-tlsr_35-3-265] Miranda AF, Kumar NR, Spangenberg G, Subudhi S, Lal B, Mouradov A (2020). Aquatic plants, *Landoltia punctata*, and *Azolla filiculoides* as bio-converters of wastewater to biofuel. Plants.

[b41-tlsr_35-3-265] Mitchell DS, Tur NM (1975). The rate of growth of Salvinia molesta (S. Auriculata Auct.) in laboratory and natural conditions. Journal of Applied Ecology.

[b42-tlsr_35-3-265] Mupenzi M, Cyprian E, Idupulapati MR, Ignatius VN (2017). Effect of cutting time on agronomic and nutritional characteristics of nine commercial cultivars of Brachiaria grass compared with Napier grass during establishment under semi-arid conditions in Rwanda. African Journal of Agricultural Research.

[b43-tlsr_35-3-265] Nasir NANM, Kamaruddin SA, Zakarya IA, Islam AKMA (2022). Sustainable alternative animal feeds: Recent advances and future perspective of using azolla as animal feed in livestock, poultry and fish nutrition. Sustainable Chemistry and Pharmacy.

[b44-tlsr_35-3-265] Pandiyan P, Sitharthan R, Saravanan S, Prabaharan N, Ramji Tiwari M, Chinnadurai T, Yuvaraj T, Devabalaji KR (2022). A comprehensive review of the prospects for rural electrification using stand-alone and hybrid energy technologies. Sustainable Energy Technologies and Assessments.

[b45-tlsr_35-3-265] Parashuramulu S, Swain PS, Nagalakshmi D (2013). Protein fractionation and in vitro digestibility of Azolla in ruminants. Online Journal of Animal and Feed Research.

[b46-tlsr_35-3-265] Robidoux PY, Virginie B, Judith L, Marc D (2018). Assessment of acute and chronic toxicity of unweathered and weathered diluted bitumen to freshwater fish and invertebrates. Ecotoxicology and Environmental Safety.

[b47-tlsr_35-3-265] Samad FA, Idris LH, Abu Hassim H, Goh YM, Loh TC (2020). Effects of Azolla spp. as feed ingredient on the growth performance and nutrient digestibility of broiler chicken. Journal of Animal Physiology and Animal Nutrition.

[b48-tlsr_35-3-265] Saunders RMK, Fowler K (1992). A morphological taxonomic revision of Azolla Lam. section Rhizosperma (Mey.) Mett. (Azollaceae). Botanical Journal of the Linnean Society.

[b49-tlsr_35-3-265] Schader C, Muller A, Scialabba NEH, Hecht J, Isensee A, Erb KH, Smith P, Makkar HP, Klocke P, Leiber F, Schwegler P (2015). Impacts of feeding less food-competing feedstuffs to livestock on global food system sustainability. Journal of the Royal Society Interface.

[b50-tlsr_35-3-265] Shukla M, Bhattacharyya A, Shukla PK, Roy D, Yadav B, Sirohi R (2018). Effect of Azolla feeding on the growth, feed conversion ratio, blood biochemical attributes and immune competence traits of growing turkeys. Veterinary World.

[b51-tlsr_35-3-265] Sireesha A, Chakravarthi MK, Naveen Z, Naik BR, Babu PR (2017). Carcass characteristics of New Zealand white rabbits fed with graded levels of Azolla (*Azolla pinnata*) in the basal diet. International Journal of Livestock Research.

[b52-tlsr_35-3-265] Soest PV (1963). Use of detergents in the analysis of fibrous feeds. I. Preparation of fiber residues of low nitrogen content. Journal of the Association of Official Agricultural Chemists.

[b53-tlsr_35-3-265] Soest PV (1967). Development of a comprehensive system of feed analyses and its application to forages. Journal of animal Science.

[b54-tlsr_35-3-265] Stafford PJ (2003). AZOLLACEAE. Review of Palaeobotany and Palynology.

[b55-tlsr_35-3-265] Stewart RM, Boyd WA (1999). The grass carp stocking rate model (AMUR/STOCK). Aquatic Plant Control Technical Note MI-03.

[b56-tlsr_35-3-265] Tallentire CW, Mackenzie SG, Kyriazakis I (2018). Can novel ingredients replace soybeans and reduce the environmental burdens of European livestock systems in the future?. Journal of Cleaner Production.

[b57-tlsr_35-3-265] Unadkat K, Parikh P (2017). A review on heavy metal absorption capacity of aquatic plants: Sources, impact and remediation technique. International Peer Reviewed Refereed Journal.

[b58-tlsr_35-3-265] Williams PH, Haynes RJ (1995). Effect of sheep, deer and cattle dung on herbage production and soil nutrient content. Grass and Forage Science.

[b59-tlsr_35-3-265] Xie T, Reddy KR, Wang C, Yargicoglu E, Spokas K (2015). Characteristics and applications of biochar for environmental remediation: A review. Critical Reviews in Environmental Science and Technology.

[b60-tlsr_35-3-265] Zailan MZ, Yaakub H, Jusoh S (2016a). In vitro digestibility and gas production characteristics of four Napier (*Pennisetum purpureum*) cultivars as fresh fodder. Malaysian Journal of Animal Science.

[b61-tlsr_35-3-265] Zailan MZ, Yaakub H, Jusoh S (2016b). Yield and nutritive value of four Napier (*Pennisetum purpureum*) cultivars at different harvesting ages. American Journal of Agricultural and Biological Science.

